# Supplementary material for: Condensin locates at transcriptional termination sites in mitosis, possibly releasing mitotic transcripts
Source: Open Biol. 2019 Oct 16;9(10):190125. doi: 10.1098/rsob.190125 (PMC6833218; doi:10.1098/rsob.190125)
Supplement: Electronic supplementary material [file rsob190125supp1.pdf]

Electronic supplementary material
Open Biology, DOI:10.1098/rsob.190125

Condensin locates at transcriptional termination sites in mitosis, possibly releasing mitotic transcripts

Norihiko Nakazawa[*,1], Orie Arakawa, and Mitsuhiro Yanagida[*]

Okinawa Institute of Science and Technology Graduate University, G0 Cell Unit, Onna-son, Okinawa, Japan 904-0495.

* Corresponding authors: nakazawa@oist.jp, myanagid@gmail.com
[1] Present address: Kochi University of Technology, School of Environmental Science and Engineering, Life Science and Technology Course, Chromosome Function and Regulation Laboratory, 185 Miyanokuchi, Tosayamada-cho, Kami, Kochi, 782-8502, Japan, Email: nakazawa.norihiko@kochi-tech.ac.jp

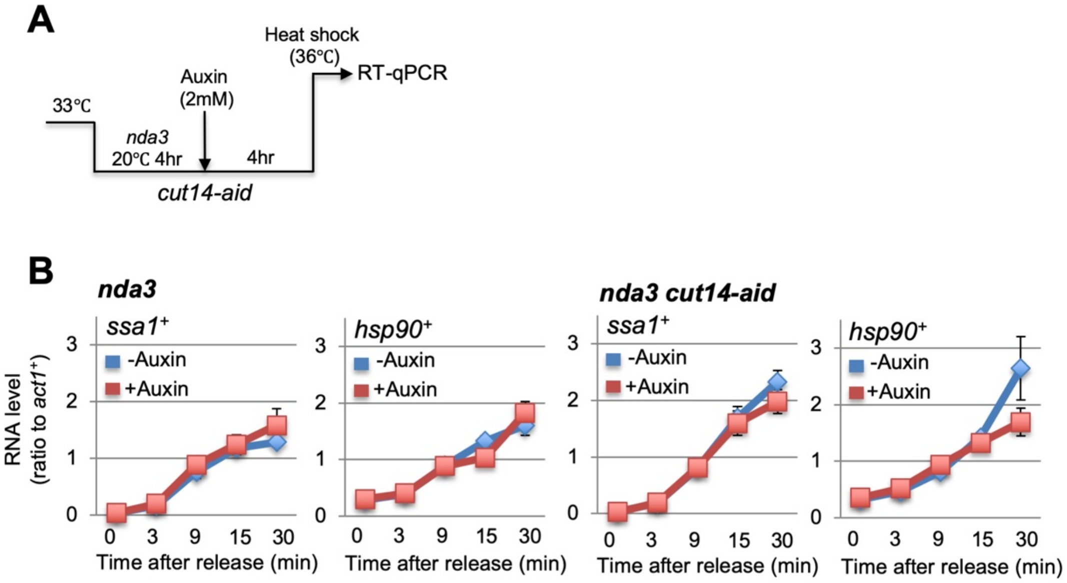

Supplemental Figure S1 Transcriptional induction was not affected in the *cut14-aid* strain (RT-qPCR confirmation of Figure 2B)
(A) Procedures for Cut14 degradation in mitotically arrested cells and transcriptional induction of heat shock-inducible genes (hsp genes). **(B)** Reverse transcriptional-quantitative PCR (RT-qPCR) was performed using primers specific to the *ssa1*$^{+}$ and *hsp90*$^{+}$ genes. In *nda3 cut14-aid* mutant cells, there is no significant change of transcriptional induction of hsp genes between Cut14-intact (-auxin) and Cut14-degraded (+auxin) condition.

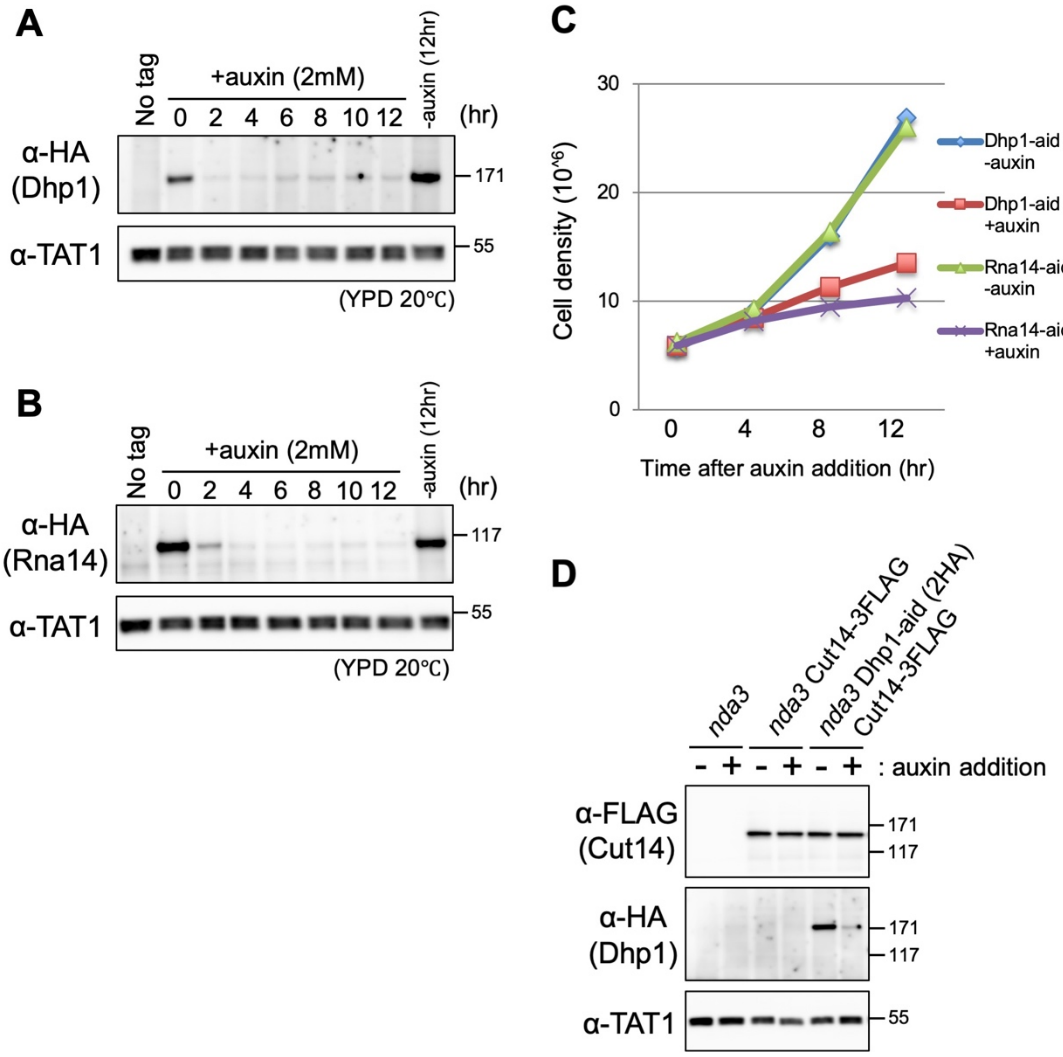

Supplemental Figure S2 Auxin-induced degradation of Dhp1 and Rna14
(A) The level of Dhp1-2HA-aid protein was assayed in the presence or absence of auxin by immunoblot using antibody against HA. Cells expressing HA- and IAA17 peptide-tagged Dhp1 with skp1-AtTIR1-NLS proteins were cultured at 20° C in the presence or absence of auxin (2 mM) for indicated periods (hr). Cells expressing only skp1-AtTIR1-NLS were used as negative controls in the presence of DMSO (No tag). Proteins in whole-cell extracts were separated with 3-8% Tris-Acetate gel electrophoresis (NuPAGE) and analyzed by immunoblotting with anti-HA and TAT1 (tubulin) antibodies. **(B)** Levels of Rna14-2HA-aid protein in the presence or absence of auxin were analyzed as shown in (A). **(C)** Cell densities of *dhp1-* and *rna14-aid* strains with or without auxin were measured at the indicated times. **(D)** The amount of Cut14-3FLAG protein was analyzed in *dhp1-aid* cells with or without auxin, as shown above. Protein levels of Cut14-FLAG were not affected by degradation of Dhp1.

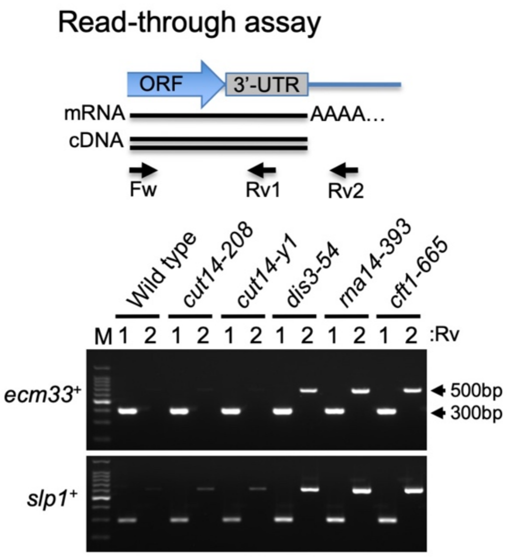

Supplemental Figure S3 Transcriptional termination was not affected in condensin ts mutants

Transcriptional read-through assays were performed in condensin ts mutants, *cut14-208* and *cut14-y1*. (Top) Schematic representation of the read-through assay. Total RNA was extracted, and then cDNA was reverse-transcribed by RT-PCR. Abnormally extended RNA product was amplified using a Fw-Rv2 primer set, in addition to PCR products with a Fw-Rv1 set. (Bottom) As performed in Figure 2E, RT-PCR products from wild-type, *cut14-208*, *cut14-y1*, *dis3-54*, *rna14-393*, and *cft1-665* mutant cells were separated in a 1.5% agarose gel. Wild-type, *cut14-208*, and *cut14-y1* were incubated at 36° C for 1.5 hr. *rna14-393* and *cft1-665* mutants were incubated at 36° C for 3 hr. The *dis3-54* cs mutant was incubated at 20° C for 8 hr. mRNA of M-phase upregulated genes (*ecm33*$^{+}$ and *slp1*$^{+}$) was amplified by RT-PCR. Lane numbers indicate reverse primer (Rv1 or 2) used in RT-PCR. M: 100-bp ladder size marker. *cut14-208* and *cut14-y1* mutants did not produce extended transcriptional products, unlike those in *dis3-54*, *rna14-393*, and *cft1-665* mutant cells.
